# Preliminary Evaluation of a NitrAdine-Based Brushing Solution for Patients Suffering from Gingivitis: A Prospective Clinical Case–Control Study

**DOI:** 10.1055/s-0041-1741120

**Published:** 2021-12-07

**Authors:** Michele Perelli, Roberto Abundo, Mario Semenza, Mauro Centracchio, Stefano Di Chiara, Andrea Monaco, Paolo Giacomo Arduino

**Affiliations:** 1 Departemnt of Periodontology, Private Practice, Torino, Italy; 2 Departemnt of Prosthodontist, Private Practice, Sant'Angelo Lodigiano, Italy; 3 Departemnt of Dental Hygiene, Private Practice, Torino, Italy; 4 Departemnt of Dental Hygiene, Private Practice, San Remo, Italy; 5 Department of Surgical Sciences, CIR-Dental School, University of Torino, Torino, Italy

**Keywords:** PerioTabs, open-label, gingivitis, outcome

## Abstract

**Objectives** This article aimed to evaluate the clinical efficacy of a nonantibiotic biofilm-removal formulation based on NitrAdine (PerioTabs), combined with a regular home oral hygiene regimen, in Caucasian patients with gingivitis.

**Materials and Methods** A sample of 60 patients were included in this clinical prospective study. All selected subjects underwent regular prophylaxis and professional oral hygiene at baseline; 30 days later, they were recalled for the measurements of the reference parameters about bleeding on probing (full-mouth bleeding upon probing score [FMBS]) and plaque index (full-mouth plaque score [FMPS]); no other clinical procedure was performed. Consequently, half of the patients ( *n* = 30) were instructed to use PerioTabs for 10 days. The remaining patients ( *n* = 30) were used as the negative control, only instructed to continue with their usual oral hygiene regimen. Fifteen days after, the clinical parameters of FMBS and FMPS were re-evaluated in both groups.

**Results** Changes in the scores of clinical indices FMBS and FMPS were calculated and compared. A significant difference between pre- and post-values, for both FMBS and FMPS, was noticed in the test group; in particular, the bleeding index value demonstrated the more significant changes: 22 participants showed a clinically meaningful improvement, and 5 had a small improvement. Only three patients had no evidence of change. In addition, 50% of patients had a reduction in plaque levels. No side effects were reported.

**Conclusions** The adjunctive use of 10-day PerioTabs treatment in the daily oral hygiene routine seemed to be efficient in reducing gingival bleeding and plaque accumulation, with absence of adverse effects. These results should be confirmed in studies with a larger number of participants following a controlled-blinded design.

## Introduction

Gingivitis is defined as a reversible inflammatory condition that affects soft support dental tissues and shows spontaneous and/or induced bleeding, gingival hypertrophy, and edema, without deep periodontal involvement. 1 2 It has multifactorial etiology, characterized by bacterial plaque and the interaction of three main cofactors: host susceptibility, environmental, and behavioral factors. 3 4 5 Bacteria and microbial biofilm (plaque) are the most important etiological agents for gingivitis. 3 4 5 Premature alteration of the gingival health status in plaque-induced gingivitis is not clinically evaluable; however, with the progression of the gingivitis, clinical signs and symptoms become more evident, having an acute or chronic development. 6 7

Plaque control and efficient plaque removal are the two key-stones for maintaining a proper health of gingival tissues; daily plaque accumulation can be prevented by home-based mechanical hygiene performed by means of tooth brushing, flossing, and other mechanical devices, 8 while plaque and calculus removal is performed during professional oral hygiene. 9 Efficient mechanical plaque control, however, is not so easy to obtain and many patients report difficulties in maintaining biofilm-free teeth and gums, hence the additional usage of chemical methods for plaque reduction, by means of antimicrobial agents, is widely accepted. 10 Different toothpastes, gels as well as mouthwashes, have been developed in an attempt to reduce bacterial adhesion, 11 being the efficacy of these products quite well documented in scientific literature. 12 Currently, the gold standard is chlorhexidine (CHX), most widely recommended as mouthwash, especially as an adjunct home-care treatment after routine scaling and root planing. 12 13 Despite its proven antibacterial activity, CHX has been associated with extrinsic staining and increased calculus formation. In addition, more reports of bacterial resistance to CHX are appearing and several papers describe cases of taste alteration, some of them with permanent consequences. 14 15 16 Because of our interest to search for alternatives to CHX, we engaged in a clinical evaluation of PerioTabs (bonyf AG, Vaduz, Liechtenstein), a NitrAdine-based tooth and gum brushing solution. Several studies have demonstrated the antibiofilm activity of NitrAdine. 17 18 19 20 Most recently, a clinical pilot study performed with periodontic patients demonstrated that PerioTabs induces a significant reduction in periodontal bacteria including *Porphyromonas gingivalis* , *Prevotella intermedia* , and *Aggregatibacter actinomycetemcomitans* , mainly due because their high concentration of surfactant. 21

We then hypothesized first that the 10-day use of PerioTabs, together with regular tooth brushing, could be useful in reducing plaque and gingival inflammation, and second to investigate tooth discoloration or other adverse reactions. We tested our hypothesis in a prospective, open-label study, by giving this medication in a cohort of subjects with clinically diagnosed gingivitis. We decided to perform this preliminary evaluation to test logistics and gather information prior to a larger randomized controlled trial regarding sample size, exclusion criteria, and materials needed.

## Materials and Methods

The study was approved by the Ethical Committee of CIR-Dental School, University of Turin (CIR-PO-pga2019/09); it was also registered on ISRCTN registry (#14006811).

Caucasian patients attending for the first time the SICOR Dental Private Practice, Turin, Italy, from December 2019 to May 2020, were selected for the present study. The same expert oral physician performed the baseline conventional intraoral examination (M.P.) and all the other measurements.

The inclusion criteria were: (1) a confirmed diagnosis of gingivitis (without probing attachment loss or radiographic bone loss); (2) absence of clinical attachment loss and absence of mobility; (3) having a minimum of 20 natural permanent teeth; (4) age ≥18 years; (5) no detectable oral mucosal lesions; (6) ability to complete the present clinical trial.

Exclusion criteria were: (1) inability or unwillingness to provide informed consent; (2) noteworthy psychiatric or cognitive impairment that could have influenced the domestic oral hygiene; (3) a smoking status; (4) patients with any fixed denture, removable prosthesis, or orthodontic appliance; (5) patients under antimicrobial therapy at least 1 month prior to the study; (6) previous head and neck radiotherapy; (7) pregnant or breast-feeding women; (8) patients in treatment with psychotropic drugs; (9) history of drug abuse; (10) subjects with an history of allergy for ingredients present in PerioTabs.

Different treatment options for the gingival status were discussed, and all patients submitted written informed consent. Investigations were performed in full accordance with the ethical principles of the World Medical Association Declaration of Helsinki of 1975, as revised in 2000.

A prospective case series with nonsurgical periodontal therapy was designed. All subjects were referred by their general dental practitioners with an enclosed set of standard radiographs; the latter was used to exclude any signs of alveolar bone loss or other bone diseases.

At baseline, all participants received oral hygiene instructions, followed by supra- and subgingival scaling as required (T0). Oral hygiene instructions were given by two equally experienced dental hygienists (M.C., S.D.C.), who also provided a professional hygiene session with ultrasounds and Gracey curettes, followed by polishing with rotating instruments (EMS Medical, Milano, Italy) and specific paste (Detartrine Z, Septodont, Matarò, Spain).

The brushing method was standardized for every patient 22 ; they were provided with a manual medium toothbrush (Curasept medium CS 820, Curadent Healthcare, Saronno, Italy), and advised to place bristles at a 45° angle to the tooth surface at the gum edge and move bristles back and forth in short (tooth-wide) strokes or small circular movements; they have to brush teeth for 120 seconds. For interdental plaque removal, they were also advised to use a dental floss (Periofloss Curaprox, Curadent Healthcare, Saronno, Italy). No indications about with type of toothpaste to use were given.

Calibration of the examiners involved doubled full-mouth probing depth measurements on 10 nonstudy subjects. The examiners were judged to be reproducible if 90% of measurements were within 1 mm of agreement between them. 22

Pictures of the oral cavity were taken at each appointment.

Four weeks after T0, subjects were recalled (T1) to receive a comprehensive periodontal examination including: full-mouth plaque scores (FMPSs) after using disclosing solution (Gum Red-Cote Plate Detector, Sunstar Butler, Mölndal, Sweden) and full-mouth bleeding upon probing scores (FMBSs), as previously reported. 22 All clinical periodontal measurements were performed on six surfaces on each tooth (mesio-buccal, mid-buccal, disto-buccal, mesio-lingual, mid-lingual, and disto-lingual), using a periodontal probe (PCPUNC15: Hu-Friedy, Chicago, Illinois, United States) by a single calibrated examiner (M.P.). 23 Stain was recorded at the buccal surface of the four upper incisors, and pigmentation was evaluated as previously reported. 24 A standardized digital sheet was used for systematic reporting of the stated parameters.

Consequently, patients were divided in two groups; the first 30 patients were enrolled in the treatment group, the other following 30 as the control group. Each participating patient in the test group received one PerioTabs box, containing 10 small effervescent tablets and a container, and the proper instruction in accordance with the manufacturer's indications. Every evening, a fresh brushing solution was prepared (by dissolving 1 small tablet in 15 mL of warm water using the provided container; while the tablet was dissolving, the toothbrush must be immersed in the solution and left for 15 minutes [the time required for the tablet to completely dissolve]). Patients then brushed their teeth and gums (inner and outer) with the solution for 2 to 3 minutes. It was recommended to immerse the toothbrush two to three times in the solution for a few seconds and brush again. After 2 minutes of brushing, patients were asked to rinse thoroughly their mouth with water and discard the remaining solution. No toothpaste or mouth rinse solution was used after the PerioTabs brushing session. The next morning, patients performed their routine daily oral hygiene by use of regular toothpaste. This scheme was maintained for the 10-day course of the treatment. Patients were aware about the importance of washing hands when transferring the pill to the solution and not swallowing the solution for any reasons.

A follow-up visit was then conducted 15 days (T2) after T1, and the same clinical periodontal parameters were recorded again as end points of the study. In addition, pictures of the patients' oral cavity were taken again as well.

The primary outcome measure of the study was the change in gingival indexes (considering FMBS and FMPS) between T1 and T2. Differences of reported indexes were also classified as follow: meaningful, with a variation of more than 30%; nonmeaningful, if between 10 and 30%; no change, if less than 10% or worsening.

Reported adverse events due to treatment were considered as secondary outcomes; the patients were also provided with a diary to record treatment's unexpected effects (e.g., gastrointestinal disease, headache, dizziness, worsening of dry mouth, or anything else reported).

Cases' sample size was difficult to estimate because of the lack previously reported data of this type of therapy in patients with gingivitis. Otherwise, the sample size was calculated according to available other data on periodontitis: with a power of 92% and a type I error of 0.05, 60 patients (30 for each arm) were needed.

Demographic and clinical characteristics were described by mean and standard deviation, or median and interquartile range, or frequency and percentage, according to variable distributions. The paired sample test was used to compare scores on two different variables within the same group; the independent sample test was used to compare scores on the same variable of the two different groups. A *p* -value of less than 0.05 was considered as significant. Data description and analysis were done using the SPSS program version 25 (Armonk, New York, IBM Corp.).

## Results

Seventy-two consecutive Caucasian patients were observed during the study period. Two patients refused to be treated, six showed periodontal damage, and four did not complete the protocol because of personal choice.

A total of 60 individuals were finally included, of whom 30 were female (50%). Table 1 reports the sociodemographic characteristics and risk factors at baseline.

**Table 1:** Sociodemographic characteristics of the studied population

	Test group ( *N* = 30)	Control group ( *N* = 30)
Demographical variables		
Age (median [IQR]), y	43 [62.00–72.00]	41 [58.00–71.00]
Gender = male (%)	15 (50)	15 (50)
Married = yes (%)	18 (60)	20 (66.6)
Employed = yes (%)	25 (83.3)	27 (90)
Risk factors		
BMI (median [IQR])	21.81 [20.05–23.03]	20.99 [19.95–24.30]
Smoker = no (%)	30 (100)	30 (100)
Alcohol consumer = yes (%)	13 (43.3)	11 (36.6)

Abbreviations: BMI, body mass index; IQR, interquartile range.

In the test group, the initial (T1) mean values for FMBS and FMPS were 38.3 (± 16.6) and 29.9 (± 18.0) respectively, while, in the control group, they were 34.1 (± 15.6) and 38.2 (± 16.2) ( Table 2 ).

**Table 2:** The comparison of selected data at time 1 (T1) and T2

	T1	T2	*p* a
Test group			
Full mouth bleeding score (%)	38.3 ± 16.8	19.7 ± 5.8	<0.001
Full mouth plaque score (%)	28.9 ± 18.0	21.5 ± 13.6	<0.001
	T1	T2	
Control group			
Full mouth bleeding score (%)	34.1 ± 15.6	31.7 ± 15.2	<0.001
Full mouth plaque score (%)	38.2 ± 16.2	35.0 ± 15.2	<0.001

a Paired sample test.

For both groups, FMBS and FMPS were measured again 15 days after the start of both treatments at T2. In the control group, the FMBS decreased to 31.7 (± 15.2) and the FMPS resulted in a value of 35.0 (± 15.3). In the test group, a FMBS index of 19.7 (± 5.85) and a FMPS of 21.5 (± 13.6) were observed ( Table 1 ). For all the indexes, in both groups, it was possible to observe a statistically significant reduction ( *p* < .05). In the test group, regarding the FMBS, 22/30 patients (73.3%) had a clinically meaningful improvement, 5 (16.7%) had a small nonmeaningful improvement, and 3 (10%) showed no change. The mean difference regarding the bleeding value was 18.6 (95% confidence interval [CI]: 12.4–24.8, *p* < 0.001) ( Table 3 ). Regarding the FMPS, 14/30 patients (46.7%) had a clinically meaningful improvement, 7 (23.3%) had a small nonmeaningful improvement, and 9 (30%) showed no change, or increased plaque ( Table 2 ). The mean plaque index (PI) value posttreatment was 21.5 (13.6) with a mean difference, between T1 and T2, of 7.5 (95% CI: 2.5–12.4, *p* = 0.014) ( Table 3 ). Regarding the control group, the reduction in terms of bleeding and presence of plaque were less evident, but only the reduction of bleeding was statistically significant between the two groups ( *p* < 0.001).

**Table 3:** The comparison regarding the reduction of the periodontal indexes between the two groups

	Groups	*N*	Mean	Std. deviation	Std. error mean	*p* a
Reduction of plaque	Control	30	3.186	3.715	0.678	0.1
	Test	30	7.429	13.246	2.418	
Reduction of bleeding	Control	30	2.376	3.103	0.566	<0.001
	Test	30	18.599	16.686	3.046	

a Independent sample test..

**Fig. 1:**
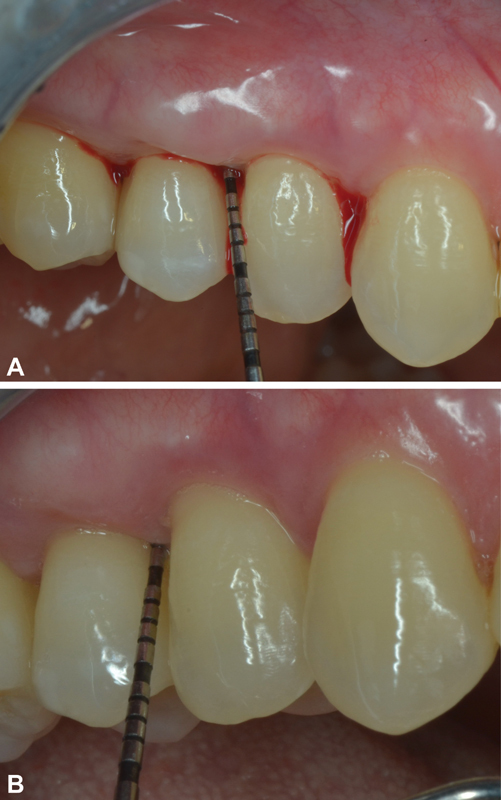
A 26-year-old woman with a diagnosed gingivitis: ( **A** ) at baseline, there is evident bleeding on probing in correspondence to tooth # 15; ( **B** ) after treatment, it is possible to see reduced probing and no bleeding in correspondence to tooth # 15.

Besides the clinical assessment of bleeding and PI, also a visual inspection of the gums after 15 days was performed and showed a well-defined improvement of the gum tissue state, in terms of color (from light red to pink), firmness (reduced edema), and attachment to the teeth ( Fig. 1 ), particularly in the test group.

No reported complications or therapy side effects were observed in any of the study individuals. The tested product did not cause hyperpigmentation. Moreover, several patients reported to have a sensation of “freshness” in their mouth and the satisfaction rate related to the use of PerioTabs was very high (personal comments of 76% of patients).

## Discussion

Different levels of gingival disease's prevention can be distinguished: primary, secondary, and tertiary prevention. 25 In particular, the primary prevention is focused on supragingival biofilm control due to the correct mechanical and /or chemical oral hygiene protocols limiting gingivitis. However, different adverse events were reported with the use of CHX or alternative products; for this reason, to overcome the side effects without loss of efficacy would be very beneficial for the patients. Moreover, the evidence of nonresponsive patients to conventional periodontal therapy highlights the need for therapeutic alternatives to treat periodontitis.

Apart from CHX, different studies are available testing topical compounds or solution for gingivitis, unfortunately with dissimilar results. To date, there has been limited evidence that curcumin could be of clinical efficacy if compared with CHX 26 ; even if with encouraging results, there is insufficient evidence to recommend the use of green tea formulation as the first-choice treatment for gingivitis 27 ; limited results have also suggested that propolis-based mouthwashes could have potential but limited benefits in reducing plaque and gingival inflammation, 28 29 as well as herbal mouthwashes. 30 More promising results have been obtained with the use of cetylpyridinium chloride mouthrinse, 31 triphala mouthwash, 32 and dentifrice-containing baking soda (BS). 33 Very limited evidence described the clinical utility for gingivitis of tea-tree oil, 34 35 hyaluronic compounds, 36 37 coenzyme Q10, 38 coconut oil, 39 and *Lippia sidoides* gel. 40

Recently, sodium hypochlorite has been used successfully as an adjunct to nonsurgical periodontal therapy; in particular, it has been demonstrated that mouthwash with 0.2% sodium hypochlorite was as effective as 0.2% CHX for the treatment of gingivitis with fewer adverse effects and less staining. 41 42

PerioTabs was delivered to introduce a new concept in providing active ingredients for oral hygiene since it is a brushing solution to be used on teeth and gums that must be prepared freshly every day. As reported, its action is based on mechanical biofilm removal using NitrAdine formula or product, a powerful nonantibiotic antibiofilm formulation in a 10-day daily brushing protocol of teeth and gums for 2 minutes. According to the manufacturer, the mode of action of the NitrAdine solution is based on the presence of a high concentration of surfactants combined with a slow release of a noncytotoxic concentration of 0.02% of sodium hypochlorite, resulting in biofilm removal and elimination of different pathogenic microorganisms. 43

In this pilot study, we have evaluated PerioTabs in 30 patients suffering from gingivitis and decided to clinically evaluate two widely used parameters, such as FMBS and FMPS, to make an initial assessment of the product's efficacy. We were able to show that a clear improvement of bleeding index value occurred in 90% of the patients after a 10-day use. In addition, 50% of the participants showed a reduced PI. It is important to note that all patients enrolled in this study underwent mechanical cleaning of the teeth 30 days prior to the initial clinical assessments, to obtain a comparable initial group.

The reduced bleeding on probing (BOP) index can be related with the specific anti-inflammatory and antibacterial role of NitrAdine; the antibacterial role, with the reducing of bacterial adhesion and eventually inactivation of bacterial behavior, has been reported as one of the basic aspects in reducing gingivitis. The anti-inflammatory capacity of the tested product is also notable because it permits to reduce or minimize the connective tissue disruption and alteration caused by all the inflammatory cascades, thus maintaining a good anatomical structure capable of healing and adhering to the tooth surface soon. Thanks to these characteristics, an inflammatory improvement of the gingival tissues has been noted, which could be useful to keep them stable over time.

In the control group, the PI improved perhaps simply thanks to the motivation given by the medical staff during the examination session; in the test group, the improvements were greater, and it is therefore possible to bring back the benefit of the solution, already in a first and immediate use.

To the best of our knowledge, the use of PerioTabs in periodontally healthy patients has never been reported. Very recently, PerioTabs was shown to be effective in reducing the gingival inflammation in patients with periodontal disease and with at least one fixed partial denture on the natural teeth. 28 Our finding, similarly to those, encouraged the use of a 10-day protocol with PerioTabs gingiva brushing solution in reducing the inflammation in healthy patients, after routine hygiene procedure.

The primary aim of this clinical pilot study was to assess the clinical efficacy of PerioTabs in a very simple protocol, but the following important limitations need to be taken into consideration: absence of a control group, no product blinding, and some participants had low BOP and PI scores limiting margin for change.

Considering the small sample size and the short follow-up, we do not want to draw definitive conclusions, but certainly the results reported could open future ways of research with a much larger study population. In conclusion, the use of PerioTabs, combined with a proper mechanical plaque control, has shown to be able to obtain a good gingival status, without any observed side effects. However, given the pilot study approach, further bigger and properly defined randomized controlled trials, with different therapeutic approaches or placebo-controlled, are needed to ascertain the real clinical effectiveness of PerioTabs for patients with gingivitis or also other periodontal conditions.
